# Supplementary material for: Dietary supplementation of alpha-lipoic acid mitigates the negative effects of heat stress in broilers
Source: PLoS One. 2021 Jul 26;16(7):e0254936. doi: 10.1371/journal.pone.0254936 (PMC8312949; doi:10.1371/journal.pone.0254936)
Supplement: Attachment — Submitted filename: Plos One review.docx [file pone.0254936.s002.docx]

Heat stress is a practical problem to broiler chicks which results in over $128 million in losses for the poultry industry in the United States. The manuscript by Wasti et al examines whether using alpha lipoic acid (ALA) mitigated negative effects of heat stress in poultry chickens. The results document the beneficial effects of ALA in broiler chickens by improving gut health. The manuscript has been written in a well-orchestrated manner. However, some points have to be discussed and manuscript could be accepted only after major revisions.

General Comments:

Line 91: Remove the space error after “phases”, and also throughout the manuscript.

Line 97: Add space between “study[9]”

Line 111: No need to explain abbreviation ADG again once it already mentioned in the abstract section.

Line 238: Dietary ALA supplementation o was able to improve the FCR. What is supplementation o? Please remove it from text.

Specific Comments:

Comment 1: Figure 1,2,3,4 No word about sample size based on which the means and SEM were computed. Please add.

Comment 2: Most of the figures do not have the superscripts. Authors have to correct those figures.

Comment 3: The exact computed P-value at least for significant ones should be shown not only (P<0.05) which is a theoretical value.

Comment 4: Can authors please describe more for antioxidant defence system and its significance in poultry?

Comment 5: Please elaborate the ALA mechanism in improving growth performance of heat stress chickens in the discussion section.

Comment 6: Authors have not mentioned any dehydration-related parameters which is the most important factor to study the heat stress-related study as this causes dehydration in animals.

Comment 7: During dehydration, mainly epithelial cells hampered. Tight junction plays a major role during heat stress. The authors focused on the claudin and occludin molecules. Results showing that insignificant changes in claudin molecule but occludin is significantly higher in the treatment group. Tight junction maintains by both of these molecules. It is assuming from this result that due to heat stress, epithelial cells in the HS group individuals may experience dehydration condition which may lead to the loss of experimental animals. Is there so? If no, then how it will be justified? However, how it will be justified about the findings of villus height (higher in NHS), crypt depth (higher in NHS), and villus surface area (same in HS and ALA+HS) in relation to claudin and occludin level? Meantime, IL-4 is reduced which is an anti-inflammatory marker in the HS group.

Comment 8: Insert superscripts in Heat shock protein-related graph (Figure 2A). How it is possible the same level of HSP70 in the NHS and HS groups? It seems that the protein folding process will be the same in the control and HS group of animals. Then how HSP90 levels get reduced in the HS group?

Comment 9: Peroxiredoxin level showing different levels in the graph but it is showing the same superscripts? Why?

Comment 10: Most of the antioxidant-related parameter levels are higher in the treatment group. Meantime SOD1 and SOD2 are higher in the NHS group. How it will be justified?
