# Supplementary material for: Dietary supplementation of alpha-lipoic acid mitigates the negative effects of heat stress in broilers
Source: PLoS One. 2021 Jul 26;16(7):e0254936. doi: 10.1371/journal.pone.0254936 (PMC8312949; doi:10.1371/journal.pone.0254936)
Supplement: Attachment — Submitted filename: Reviewer report revision 1.docx [file pone.0254936.s004.docx]

Reviewer Comments: In revising manuscript authors have done a good job in addressing all the comments raised earlier and I strongly recommended manuscript to be accepted in PLOS ONE.
