# Supplementary material for: Dietary supplementation of alpha-lipoic acid mitigates the negative effects of heat stress in broilers
Source: PLoS One. 2021 Jul 26;16(7):e0254936. doi: 10.1371/journal.pone.0254936 (PMC8312949; doi:10.1371/journal.pone.0254936)
Supplement: Attachment — Submitted filename: Response to Reviewer_PONE-D-21-05713R2.docx [file pone.0254936.s005.docx]

**Reviewer #1**

More specifically, the calcium and phosphorus values are not formulated according to the commercial birds’ recommendation (in this case Cobb 500). So, the authors answer is not correct. It is true that there is variation in the inclusion level of calcium and phosphorus. The authors must mention in the manuscript that they formulated a low calcium and phosphorus diet and if possible, should provide the reason for such formulation. Low Ca and P do not necessarily keep the birds safe. This is not going to affect the outcome of this study but would provide a wrong reference for other researchers that low Ca and P diet would be required to yield a standard growth performance. The authors should provide correct information. They cannot cite one reference for meeting requirement and provide reason that it is outdated, and they considered commercial recommendation, surprisingly which was even not followed. Cobb guidelines state Ca requirement to be 0.9% in the starter and 0.8% in the finisher, available phosphorus is around 0.45%. The authors have formulated the diet containing 0.75% Ca in the starter and 0.52% in the finisher, while the available phosphorus is 0.3% in the starter and 0.23% in the finisher. There is a typo: it should be available phosphorus and not dig. If the authors provide correct information in the draft and state the reason for this inclusion level then it would be ok otherwise it would be understood as a mistake in their feed formulation.

Response: We highly appreciate the reviewer’s meticulous review and pointing out about the feed formulation (following the recent commercial standard). Yes, we agree with the reviewer’s point that we have used lower amount of the calcium and phosphorus than that is recommended for the commercial requirements. We have added this information in our revised manuscript. (Line 94-95). The objective of this study is focused on mitigating heat stress using nutritional supplementation (Alpha lipoic acid). So, our focus was on meeting the energy and protein requirement following the NRC (1994) guidelines and examining at the variables related to heat stress. We agree with the reviewer that we should have formulate diets based on commercial requirement, which we believe is a valuable input for executing our future projects. In this study, however, we believe that lower Ca and P level will not affect the tested variable. So, the finding of the study still holds scientific merits as it provides valuable information on mitigation of heat stress.

Corrections:

- Birds were fed the corn-soybean meal-based diets in two phase, starter (1-21d) and finisher (22-42d). The energy and protein requirements of the diet was met following the NRC (1994); however, Ca and P level was used at lower level than the commercial requirements (Line 94-95).
- We have corrected “dig-P” to “available phosphorous” (in the Table-1, Line 102)

Again, thank you so much for your critical review for the refinement of this manuscript, and we will follow the recent NRC guidelines in our future study as per reviewer’s recommendation.

2. Also, authors should have removed 'standard curve method' from line 146 if they calculated gene expression by the comparative CT method. Figure 2 does not show any fold change (as stated in line 147) as none of the treatment has a normalized value of 1. So, it would be better to remove the fold change from the materials and methods. Besides, the authors should mention the treatment (may be the control group) that was used to calculate the ΔΔ CT.

Response: Standard curve has been removed from the method section, and was modified

In line 147-150: The expression level of target genes was determined using the cycle threshold (Ct) values and changes in the gene expression were calculated by the 2-ΔΔCt method compared to the control group. The relative mRNA expression was normalized to the endogenous reference gene *B-actin*.
